# Electric field tunability of the electronic properties and contact types in the MoS_2_/SiH heterostructure†

**DOI:** 10.1039/d2ra03817j

**Published:** 2022-08-25

**Authors:** Son-Tung Nguyen, Chuong V. Nguyen, Kien Nguyen-Ba, Huy Le-Quoc, Nguyen V. Hieu, Cuong Q. Nguyen

**Affiliations:** Faculty of Electrical Engineering, Hanoi University of Industry Hanoi 100000 Vietnam; Department of Materials Science and Engineering, Le Quy Don Technical University Hanoi Vietnam; The University of Danang – University of Science and Technology Danang 550000 Vietnam lqhuy@dut.udn.vn; Physics Department, The University of Danang – University of Science and Education Da Nang 550000 Vietnam; Institute of Research and Development, Duy Tan University Da Nang 550000 Vietnam nguyenquangcuong3@duytan.edu.vn; Faculty of Natural Sciences, Duy Tan University Da Nang 550000 Vietnam

## Abstract

The generation of layered heterostructures with type-II band alignment is considered to be an effective tool for the design and fabrication of a highly efficient photocatalyst. In this work, we design a novel type-II MoS_2_/SiH HTS and investigate its atomic structure, electronic properties and contact types. In the ground state, the MoS_2_/SiH HTS is proved to be structurally and mechanically stable. The MoS_2_/SiH HTS generates type-II band alignment with separation of the photogenerated carriers. Both the electronic properties and contact type of the MoS_2_/SiH HTS can be modulated by an external electric field. The application of a negative electric field leads to a transformation from type-II to type-I band alignment. While the application of a positive electric field gives rise to a transition from semiconductor to metal in the MoS_2_/SiH HTS. These results could provide useful information for the design and fabrication of photoelectric devices on the MoS_2_/SiH HTS.

## Abstract 2

The generation of layered heterostructures with type-II band alignment is considered to be an effective tool for the design and fabrication of a highly efficient photocatalyst.

## Graphical abstract

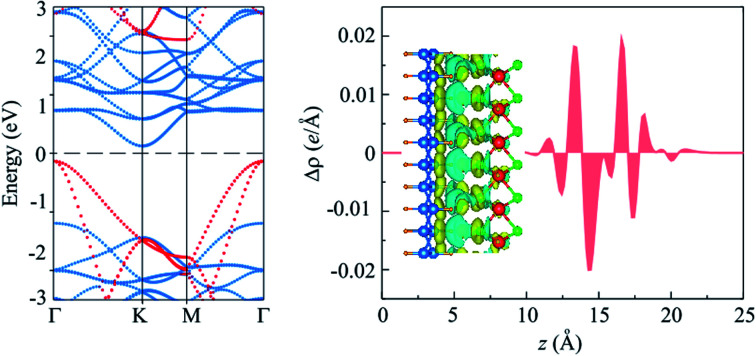

## 1 Introduction

The successful isolation of graphene^1^ has opened up a new research direction in novel materials science. Over nearly one decade, a series of two-dimensional materials^2–4^ have been discovered, synthesized experimentally and investigated systematically. 2D materials are proved to have outstanding physical and chemical properties that make them promising candidates for various applications, including transistors,^5^ Li-ion batteries,^6^ water splitting^7^ and gas sensors.^8^ Graphene,^1^ hexagonal boron nitride (h-BN),^9^ transition metal dichalcogenides (TMDs)^10^ and phosphorene analogues^11,12^ are currently considered to be the most attractive 2D materials owing to their intriguing properties and wide range of applications. Although these 2D materials have promising properties, they also exhibit some disadvantages that may limit their applications in various fields. For instance, the lack of band gap in graphene^13^ limits its application in field-effect transistors.^14^ Unlike graphene, phosphorene is a semiconductor with a finite band gap. However, the structural instability under ambient conditions of phosphorene limits its application in modern day devices.^15^ Therefore, the search for novel 2D materials as well as finding common approaches to control their properties for various applications is still challenging.

Currently, one of the most commonly used techniques to improve the properties and expand the application range of 2D materials is the generation of van der Waals (vdW) heterostructures (HTSs) between two or more different 2D materials.^16,17^ The different 2D materials in their vdW-HTSs are held together by the weak vdW forces. Thus, the intrinsic excellent properties of the constituent 2D materials are maintained in their combined HTSs. Moreover, the formation of vdW-HTSs between 2D materials may also give rise to the creation of novel properties that may not exist in the constituent 2D monolayers. Generally, the recombination of the photogenerated carriers in single-layer 2D materials is very rapid. Whereas, the photogenerated carriers in the combined vdW-HTSs between different 2D materials are separated effectively depending on the positions of the band edges of the constituent monolayers. The combination between two different 2D materials results in the formation of type-I, type-II or type-III band alignment, as depicted in Fig. S1 of the ESI.† For type-I, the conduction band minimum (CBM)/valence band maximum (VBM) of one layer is higher/lower than that of the other layer. In type-II, the CBM and VBM of the heterostructure come from different constituent monolayers. In type-III, the CBM of one layer is lower than the VBM of the other layer. To date, a large number of HTSs have been formed between two or more different 2D materials, such as graphene HTSs^18–21^ and TMD HTSs.^22–25^ The combination between two or more different 2D materials may also give rise to the appearance of novel properties that may not be observed in the 2D monolayers.

Recently, a novel 2D material, namely silane (SiH), was obtained by the covalent modification of hydrogen and silicene.^26,27^ Unlike silicene, the SiH monolayer is a semiconductor with a band gap of about 2.19 eV,^28^ making it suitable for photocatalysis and optoelectronic applications.^29,30^ The SiH monolayer is structurally stable at room temperature. The combination between the SiH monolayer and other 2D materials has been proposed and predicted recently. For instance, Han *et al.* investigated type-II band alignment in the GaAs/SiH HTS using first principles calculations.^31^ The results showed that the formation of the type-II GaAs/SiH HTS leads to an enhancement of the optical absorption in the visible light region. Zeng *et al.*^29^ constructed a novel SiH/CeO_2_(111) HTS and investigated its electronic and optical properties and photocatalytic performance. The results demonstrated that the novel SiH/CeO_2_(111) HTS generates type-II band alignment and it is a promising photocatalyst for splitting water to hydrogen. All the above-mentioned findings suggest that the SiH monolayer can be used to form HTSs with other 2D materials. Currently, MoS_2_ is one of the most attractive materials in the 2D TMD family.^32^ HTSs between MoS_2_ and other 2D materials have not only been predicted theoretically^33,34^ but also synthesized experimentally.^35–37^ However, to date, the combination between single layer SiH and MoS_2_ monolayers has not yet been designed and investigated.

In this work, we perform first principles calculations to design a novel MoS_2_/SiH HTS and investigate its atomic structure, electronic properties and contact types. Our results show that the MoS_2_/SiH HTS is structurally and mechanically stable in the ground state. The MoS_2_/SiH HTS generates type-II band alignment, making it a promising candidate as an efficient photovoltaic device because the photogenerated carriers in type-II are separated in the two materials. Both the electronic properties and contact type of the MoS_2_/SiH HTS can be modulated by an external electric field. The application of a negative electric field leads to a transformation from type-II to type-I band alignment. While the application of a positive electric field gives rise to a transition from semiconductor to metal in the MoS_2_/SiH HTS. These results could provide useful information for the design and synthesis of photocatalytic devices based on the MoS_2_/SiH HTS.

## 2 Computational methods

All calculations are performed in the framework of density functional theory (DFT) using first-principles calculations.^38^ The Vienna *ab initio* simulation (VASP)^39,40^ is used to perform the structural optimization and electronic properties prediction. The Perdew–Burke–Ernzerhof (PBE) functional in the framework of the generalized gradient approximation (GGA)^41,42^ is used to describe the exchange–correlation force. The HSE06 functional is chosen to obtain more accurately the band gaps of the materials.^43^ A vacuum space of 20 Å is used to avoid all unnecessary neighboring layered interactions in materials. A *k*-point mesh of 12 × 12 × 1 and a cut-off energy of 510 eV are chosen. All structural geometries are fully optimized until the convergence tolerance of energy and force which are less than 10^−6^ eV and 10^−3^ eV Å^−1^, respectively. A DFT-D3 method^44^ is also adopted to describe the long-range forces in the layered 2D materials.

## 3 Results and discussion

**Fig. 1:**
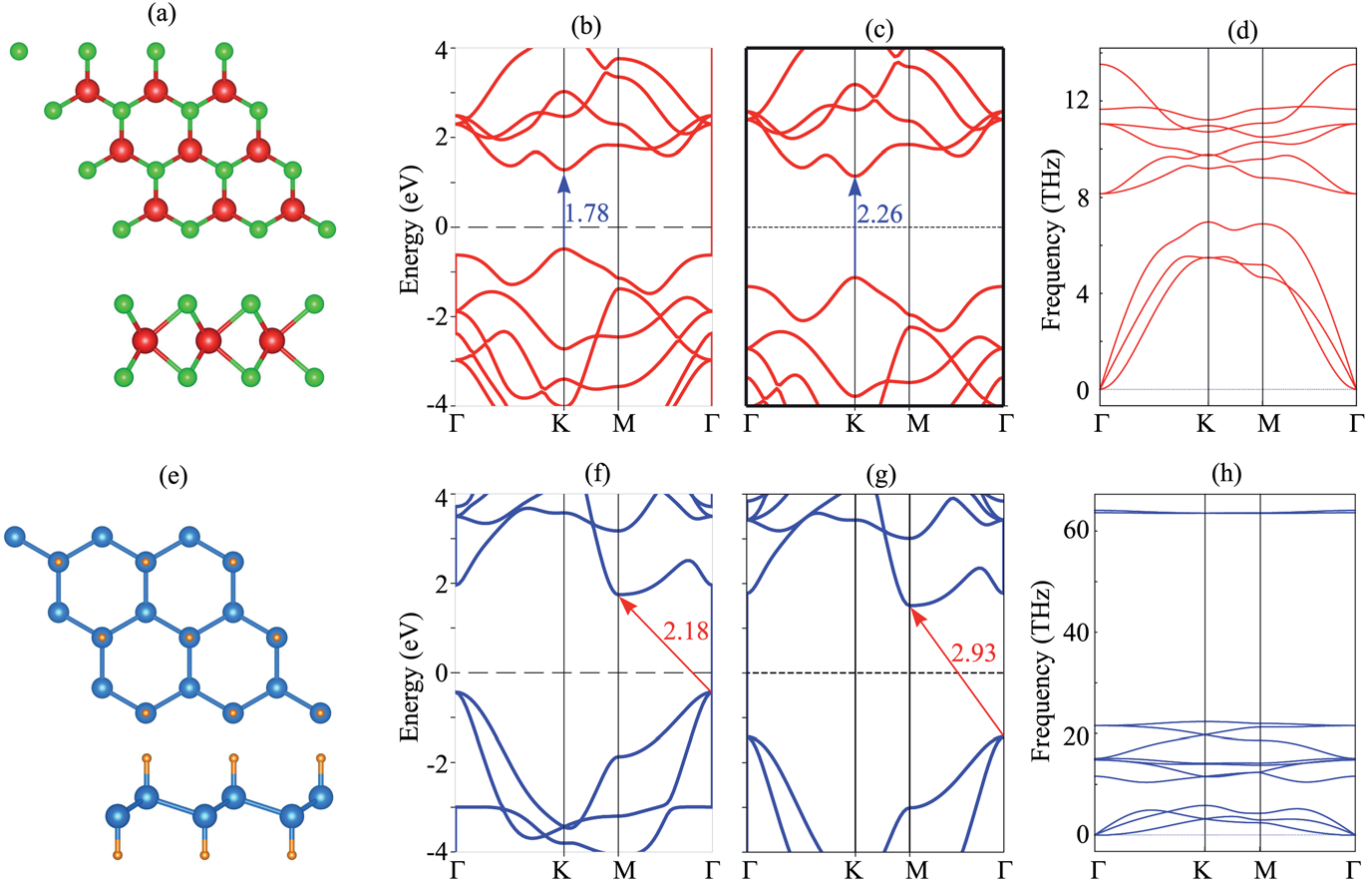
(a and e) The relaxed atomic structures, (b and f) PBE band structure, (c and g) HSE band structure and (d and h) phonon spectrum of the MoS_2_/SiH layer.

We first investigate the atomic structure and electronic properties of MoS_2_ and SiH monolayers. The atomic structures of these monolayers are depicted in Fig. 1(a) and (e). The lattice constants of MoS_2_ and SiH monolayers after the geometric optimization are calculated to be 3.18 and 3.86 Å, respectively. These values are in good agreement with the previous reports.^26,29,45–47^ The band structures of MoS_2_ and SiH monolayers obtained by the PBE and HSE functionals are depicted in Fig. 1(b), (c), (f) and (g). Both the MoS_2_ and SiH monolayers are semiconductors with the band gap values of 1.78/2.26 and 2.18/2.93 eV obtained with the PBE/HSE functional, respectively. The MoS_2_ monolayer possesses a direct band gap with conduction band minimum (CBM) and valence band maximum (VBM) at the K point. Whereas, the SiH monolayer exhibits an indirect band gap with the VBM at the Γ point and the CBM at the M point. Both the PBE and HSE functionals predict the same characteristics of MoS_2_ and SiH monolayers, suggesting that the PBE method predicts the correct trends and physical mechanisms for the MoS_2_/SiH heterostructure. Thus, we decide to choose the PBE functional for all the next calculations because of a low computational cost. Moreover, our goal is not to acquire the precise band gaps of the MoS_2_/SiH heterostructure, but to explore the trend in electronic properties and contact types under external factors, such as external electric field.

**Fig. 2:**
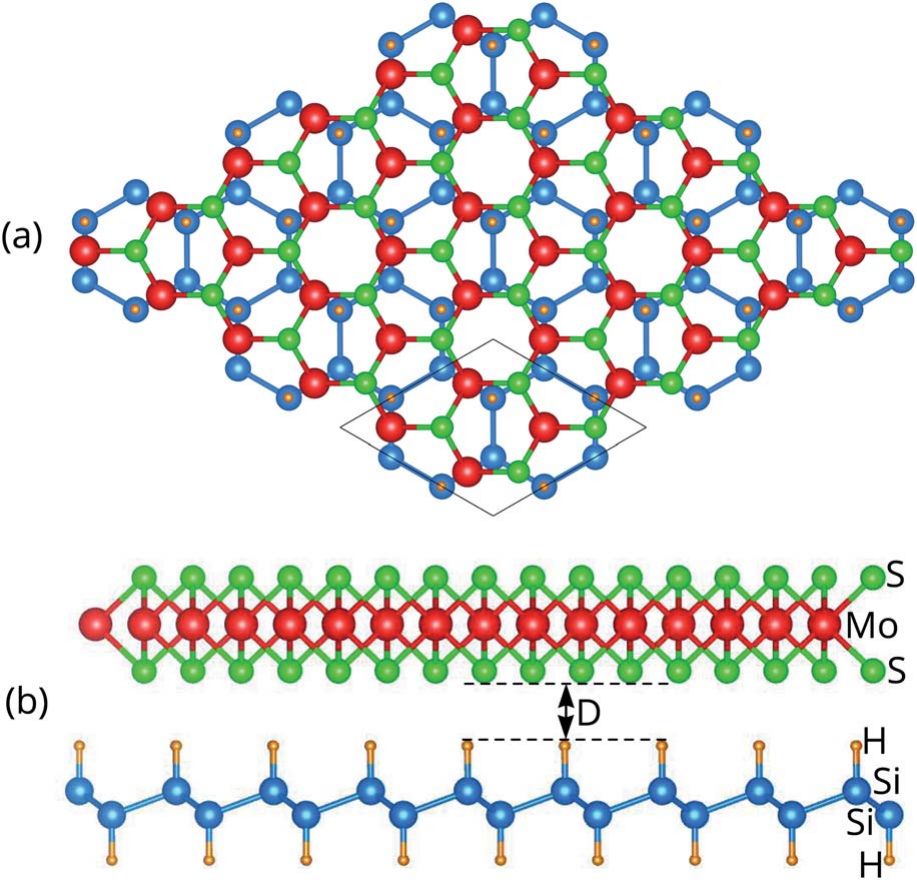
(a) Top view and (b) side view of the most energetically stable stacking configuration of the MoS_2_/SiH HTS. The supercell of the considered HTS is marked by a black line.

We next design the combination between two different MoS_2_ and SiH monolayers to form the MoS_2_/SiH heterostructure. We designed five different stacking configurations of the MoS_2_/SiH heterostructure. The most energetically stable stacking configuration of the MoS_2_/SiH heterostructure is shown in Fig. 2, while the other stacking configurations are illustrated in Fig. S2 of the ESI.† It should be noted that the stacking configuration of the MoS_2_/SiH heterostructure presented in the manuscript is the most energetically stable heterostructure because it has the lowest binding energy compared to the others. In order to design the MoS_2_/SiH heterostructure, we use a supercell, consisting of (2 × 2) unit cells of the MoS_2_ layer and 
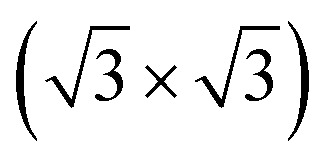
 unit cells of the SiH monolayer. The lattice constant of the MoS_2_/SiH heterostructure after geometric optimization is found to be 6.43 Å. The lattice mismatch in the MoS_2_/SiH heterostructure is calculated to be less than 2%, which is small and affects insignificantly the electronic features of the heterostructure. After geometric optimization, the interlayer spacing between MoS_2_ and SiH layers in the corresponding heterostructure is 2.48 Å. This interlayer spacing is in the same order of magnitude as other Si(Ge)H-based heterostructures, such as GeH/graphene,^48^ SiH/PtSe_2_,^47^ and SiH/AlAs.^49^ Moreover, to check the structural stability of such a heterostructure, we further calculate the binding energy as: *E*_b_ = [*E*_H_ − (*E*_M_ − *E*_S_)]/*A*, where *E*_H_, *E*_M_ and *E*_S_ are the total energies of the MoS_2_/SiH heterostructure, and isolated MoS_2_ and SiH monolayers, respectively. *A* is the surface area of the MoS_2_/SiH HTS. The binding energy of the MoS_2_/SiH HTS is −11.44 meV Å^−2^. The negative sign of the binding energy implies that the MoS_2_/SiH HTS is stable at the equilibrium interlayer spacing. Moreover, to check the mechanical stability of such a HTS, we also calculate the elastic constants of the MoS_2_/SiH HTS. The elastic constants [*C*_*ij*_] of the MoS_2_/SiH HTS include four components, namely *C*_11_, *C*_12_, *C*_22_ and *C*_66_. The MoS_2_/SiH HTS is mechanically stable when its elastic constants satisfy the Born–Huang criteria,^50^*i.e. C*_66_ > 0 and *C*_11_*C*_22_ − *C*_12_^2^ > 0. Our calculated elastic constants of the MoS_2_/SiH HTS are *C*_11_ = *C*_22_ = 188.37 N m^−1^, *C*_12_ = 48.29 N m^−1^ and *C*_66_ = 70.06 N m^−1^. These values of the elastic constants of the MoS_2_/SiH HTS indicate that it is mechanically stable.

**Fig. 3:**
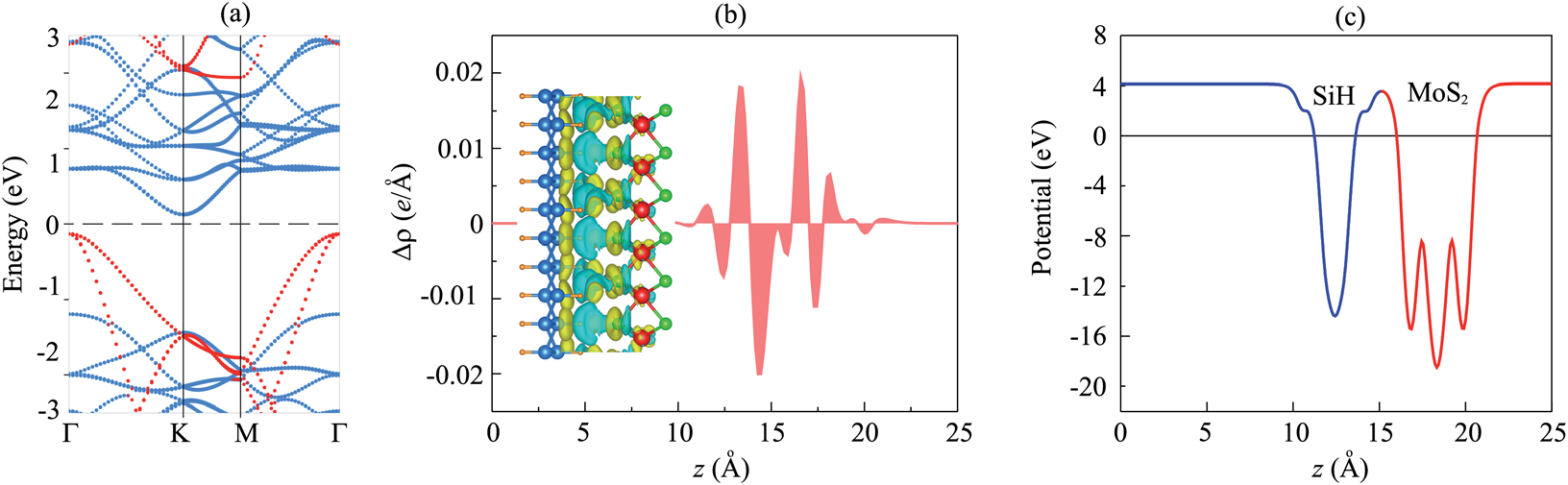
(a) Projected band structure, (b) plane-averaged charge density difference and (c) electrostatic potential of the MoS_2_/SiH HTS. The red and blue circles in (a) represent the contributions of SiH and MoS_2_ layers, respectively. The inset in (b) is the 3D charge density difference. Cyan and yellow regions represent the gain and loss of electrons, respectively.

The projected band structure of the MoS_2_/SiH HTS is illustrated in Fig. 3(a). The contributions of MoS_2_ and SiH layers are represented by blue and red circles, respectively. One can find that the MoS_2_/SiH HTS possesses a semiconducting nature with an indirect band gap of 0.26 eV, which is smaller than that of both the MoS_2_ and SiH monolayers. This finding indicates that the generation of the MoS_2_/SiH HTS gives rise to a reduction in the band gap compared to that of the constituent monolayers. More interestingly, the VBM of the MoS_2_/SiH HTS is located at the Γ point and it comes mainly from the SiH layer. Whereas, the CBM of the MoS_2_/SiH HTS is located at the M point and it is mainly contributed by the MoS_2_ layer. These contributions demonstrate that the VBM and CBM of the MoS_2_/SiH HTS come from different layers, resulting in the formation of type-II band alignment. The type-II band alignment of the MoS_2_/SiH HTS makes it a promising candidate as an efficient photovoltaic device because the photogenerated carriers in type-II are separated in the two materials.

The generation of type-II band alignment in the MoS_2_/SiH HTS may lead to charge redistribution at the interface. Therefore, we further visualize the charge density difference as Δ*ρ* = *ρ*_H_ − *ρ*_M_ − *ρ*_S_, where *ρ*_H_, *ρ*_M_ and *ρ*_S_ are the charge densities of the MoS_2_/SiH HTS, and isolated MoS_2_ and SiH monolayers, respectively. One can observe from Fig. 3(b) that there is a charge redistribution at the interface of the MoS_2_/SiH HTS. In addition, we find that the SiH layer loses electrons, while the MoS_2_ layer gains electrons. This indicates that the charges are transferred from SiH to MoS_2_ layers in the MoS_2_/SiH HTS. Furthermore, the electrostatic potential of the MoS_2_/SiH HTS in Fig. 3(c) demonstrates that the MoS_2_ layer has a deeper potential than the SiH layer, causing the creation of a built-in electric field. The potential drop between the MoS_2_ and SiH layers is 4.2 eV. It is obvious that the formation of a built-in electric field at the interface of the MoS_2_/SiH HTS gives rise to the separation of the photogenerated electrons and holes as well as the inhibition of their recombination.

Furthermore, to have a better understanding of the charge transfer between two different MoS_2_ and SiH layers, we calculate the work function of the MoS_2_/SiH HTS as well as the constituent MoS_2_ and SiH monolayers for comparison. The work function can be calculated as: *Φ* = *E*_vac_ − *E*_F_, where *E*_vac_ and *E*_F_ are the vacuum energy and the Fermi level energy, respectively. The calculated work functions of the MoS_2_/SiH HTS, and isolated MoS_2_ and SiH monolayers are 4.62, 5.15 and 5.80 eV, respectively. The difference in the work functions of the MoS_2_ and SiH monolayers allows the charge transfer from the SiH to the MoS_2_ layer. Furthermore, the work function of the MoS_2_/SiH HTS is lower than that of both the MoS_2_ and SiH monolayers, suggesting that there is a charge redistribution at the interface of the heterostructure.

**Fig. 4:**
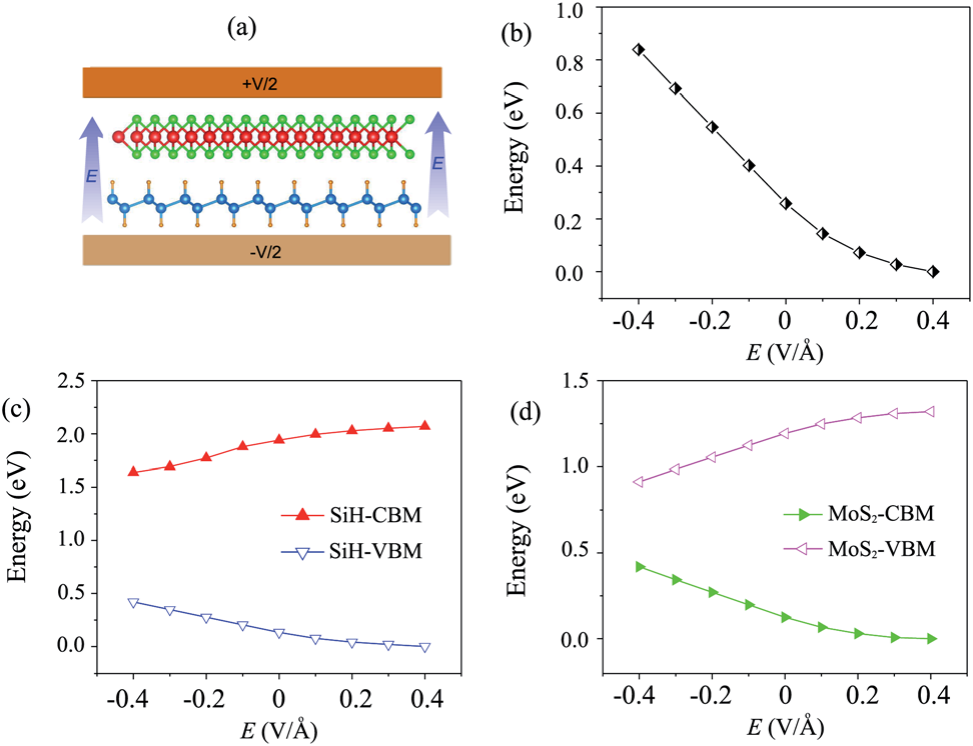
(a) Schematic model of applied-*E* to the MoS_2_/SiH HTS. The variations of (b) the band gap and the band edges of (c) SiH and (d) MoS_2_ layers as a function of electric field.

Currently, applying an electric field (*E*) is proven to be an effective tool to modify the electronic properties and contact types in the HTS between two different 2D materials. The strength of the applied *E* ranges from −0.4 V Å^−1^ to +0.4 V Å^−1^. The direction of the applied *E* is from the SiH to the MoS_2_ layer, as depicted in Fig. 4(a). It should be noted that the electric field was applied for both the optimization process and electronic properties calculations. Our results showed that the relaxed atomic structures of the MoS_2_/SiH heterostructure remain unchanged when the electric field is applied in the optimization process as compared to those without the application of the electric field, as depicted in Fig. S3 of the ESI.† The dependence of the band gap of the MoS_2_/SiH HTS as a function of applied *E* is depicted in Fig. 4(b). One can find that a positive *E* leads to a narrower band gap of the MoS_2_/SiH HTS, while a negative *E* gives rise to an increase in the band gap of the MoS_2_/SiH HTS. Under the positive *E* = +0.4 V Å^−1^, the band gap of the MoS_2_/SiH HTS is reduced to zero, indicating that the semiconducting nature in the MoS_2_/SiH HTS transforms into metallic nature. Moreover, it is obvious that the band gap changes of the MoS_2_/SiH HTS may be related to a change in the band edges of the constituent monolayers. Thus, in order to have a better understanding of the physical nature of the modulation of the band gap by the application of the *E*, we plot the positions of the VBM and CBM band edges of the constituent MoS_2_ and SiH monolayers under different values of negative and positive *E*, as illustrated in Fig. 4(c) and (d), respectively. From these figures, one can find that the CBM (VBM) value of the SiH (MoS_2_) layer is increased when the applied *E* is changed from −0.4 to +0.4 V Å^−1^. On the other hand, the VBM (CBM) of the SiH (MoS_2_) layer decreases correspondingly. As we have discussed above, type-II band alignment is formed in the MoS_2_/SiH HTS with the contribution of the SiH layer to the VBM and the contribution of the MoS_2_ layer to the CBM. The decrease in the VBM value of the SiH layer and the CBM value of the MoS_2_ layer when the applied *E* is changed from −0.4 V Å^−1^ to +0.4 V Å^−1^ leads to a narrower band gap of the MoS_2_/SiH HTS.

**Fig. 5:**
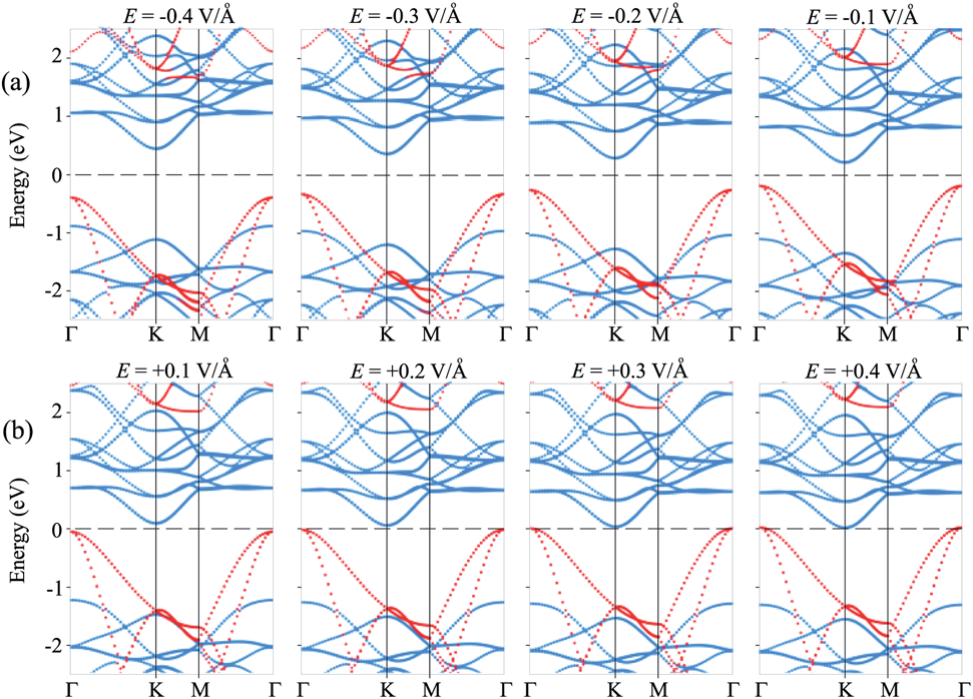
Projected band structures of the MoS_2_/SiH HTS under different values of (a) negative and (b) positive *E*. The contributions of the MoS_2_ and SiH layers are marked by blue and red circles, respectively.

Furthermore, to further comprehend the effect of electric field on the band gap of the MoS_2_/SiH HTS, we plot its projected band structures under different strengths of applied *E*, as depicted in Fig. 5. When a negative *E* is applied, the VBM (CBM) of the SiH layer shifts away from (towards) the Fermi level of the HTS. Whereas, the VBM (CBM) of the MoS_2_ layer shifts towards (away from) the Fermi level of the HTS. This trend shows that under the application of the negative *E*, the VBM of the SiH layer and the CBM of MoS_2_ shift away from the Fermi level, giving rise to an increase in the band gap of the MoS_2_/SiH HTS. Furthermore, under the application of the positive *E*, the movement of the VBM and CBM of the MoS_2_ and SiH layers in the corresponding MoS_2_/SiH HTS is in the opposite direction compared with the applied negative *E*. Thus, the positive *E* leads to a reduction of the band gap of the MoS_2_/SiH HTS. Under the application of the positive *E* = +0.4 V Å^−1^, both the VBM and CBM of the MoS_2_/SiH HTS cross the Fermi level, indicating a transformation from semiconductor to metal. Moreover, following the changing trend in the band edges of the MoS_2_ and SiH layers in the corresponding MoS_2_/SiH HTS, we predict that the VBM of the SiH layer will be lower than that of MoS_2_ at the critical strength of the negative *E* = −0.7 V Å^−1^. This prediction suggests that a transformation from type-II to type-I band alignment will be achieved under the application of a negative electric field of −0.7 V Å^−1^. However, it would not be reasonable to apply higher *E* values, since they would be rather difficult to experimentally achieve. In addition, it should be noted that to apply an electric field in devices, experimental realization is considered as one pole of the battery connecting to MoS_2_, and the other pole connecting to the SiH layer. Thus, we predict that the MoS_2_/SiH heterostructure is placed inside the capacitor configuration, from which the electric field is generated. The electric field penetrates through the whole MoS_2_ layer. The electric field outside and inside the MoS_2_/SiH heterostructure is the same when the out-of-plane dielectric polarization is calculated to be zero.

## 4 Conclusions

In conclusion, we have designed a type-II MoS_2_/SiH HTS and investigated its structural and electronic properties and the formation of the contact types by performing first principles calculations. The MoS_2_/SiH HTS is proved to be structurally and mechanically stable in the equilibrium state. The formation of the MoS_2_/SiH HTS leads to a reduction of the band gap compared to that of the constituent MoS_2_ and SiH monolayers, indicating that the electrons move quickly from the VB to the CB in such a HTS. Moreover, the MoS_2_/SiH HTS forms type-II band alignment, indicating that such a HTS is a promising candidate as an efficient photoelectric device because the photogenerated carriers in type-II are separated in each material. Furthermore, the electronic properties and contact type of the MoS_2_/SiH HTS can be modulated by an external electric field. The application of a negative electric field leads to a transformation from type-II to type-I band alignment. While the application of a positive electric field gives rise to a transition from semiconductor to metal in the MoS_2_/SiH HTS. These results could provide useful information for the design and synthesis of photoelectric devices based on the MoS_2_/SiH HTS.

## Conflicts of interest

There are no conflicts to declare.

## Supplementary Material

RA-012-D2RA03817J-s001
