# Supplementary material for: Electric field tunability of the electronic properties and contact types in the MoS2/SiH heterostructure
Source: RSC Adv. 2022 Aug 25;12(37):24172–7. doi: 10.1039/d2ra03817j (PMC9403661; doi:10.1039/d2ra03817j)
Supplement: RA-012-D2RA03817J-s001 [file RA-012-D2RA03817J-s001.pdf]

Electronic Supplementary Material (ESI) for RSC Advances.
This journal is © The Royal Society of Chemistry 2022

Supporting Information (SI)

Electric field tunable of the electronic properties and contact types in MoS2/SiH heterostructure

Son-Tung Nguyen[1], Chuong V. Nguyen[2], Kien Nguyen-Ba[3], Huy Le-Quoc[3,†], Nguyen V. Hieu[4] and Cuong Q. Nguyen[5,6,†]

[1]*Faculty of Electrical Engineering Technology, Hanoi University of Industry, Hanoi 100000, Vietnam*

[2]*Department of Materials Science and Engineering, Le Quy Don Technical University, Ha Noi 100000, Vietnam*

[3]*The University of Danang - University of Science and Technology, Danang 550000, Vietnam.*

[4]*Physics Department, The University of Danang - University of Science and Education, Da Nang 550000, Vietnam*

[5]*Institute of Research and Development, Duy Tan University, Da Nang 550000, Vietnam.*

[6]*Faculty of Natural Sciences, Duy Tan University, Da Nang 550000, Vietnam*

[†]*Corresponding authors:* lqhuy@dut.udn.vn *and* nguyenquangcuong3@duytan.edu.vn

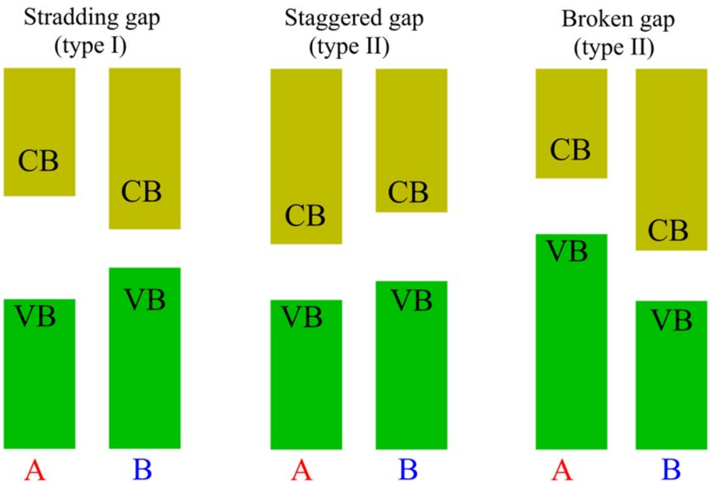

Fig. S1. Band alignments between two different semiconductors A and B.

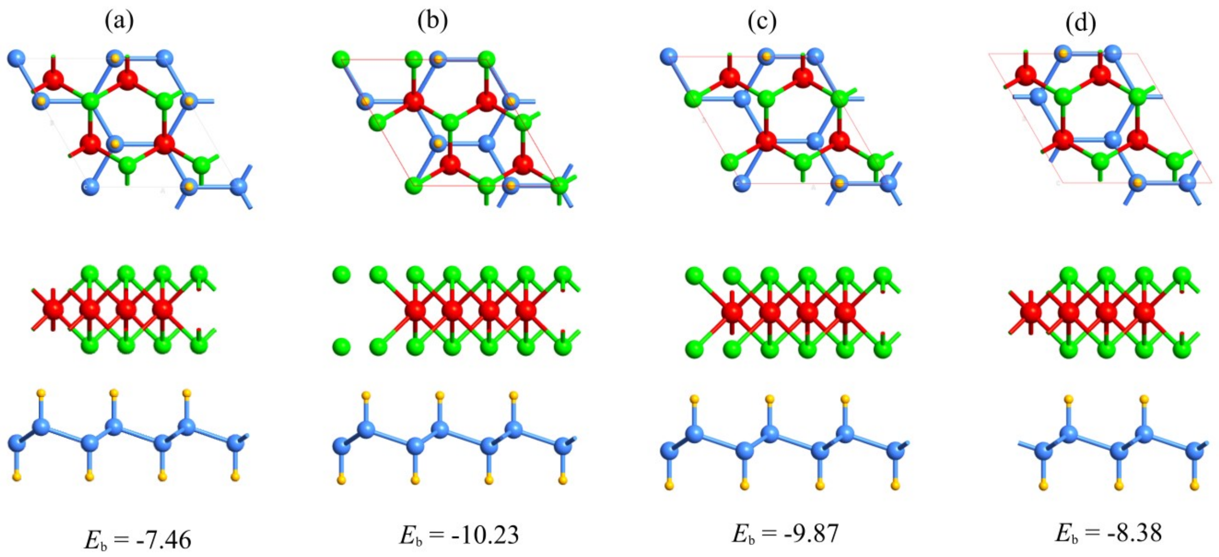

Fig. S2. Different stacking configurations of MoS2/SiH heterostructures (a) stacking I, (b) stacking II, (c) stacking III and (d) stacking IV.

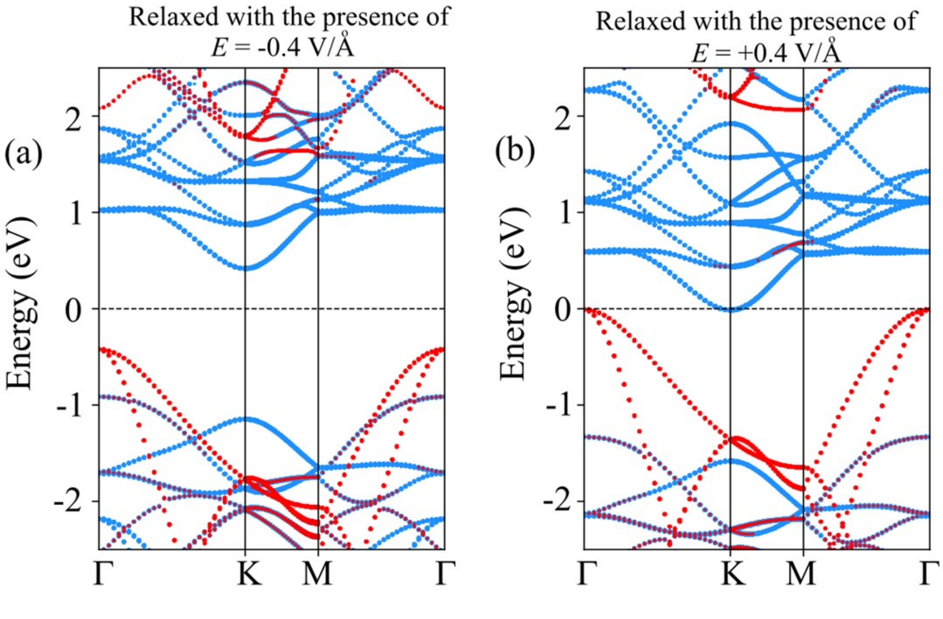

Fig. S3. Projected band structures of MoS2/SiH heterostructure under the applied strengths of electric field of (a) E = -0.4 V/Å and (b) E = +0.4 V/Å. These values of electric field were applied for both the geometric optimization and electronic properties calculations.
